# Emphysematous pyelonephritis and infection-related calculi

**DOI:** 10.1007/s00467-025-06918-8

**Published:** 2025-09-30

**Authors:** Shimrit Tzvi-Behr, Yaacov Frishberg, Ruth Cytter-Kuint, Boris Chertin, Ilan Z. Kafka, Efrat Ben-Shalom

**Affiliations:** 1 Division of Pediatric Nephrology, Shaare Zedek Medical Center, Jerusalem, Israel; 2 Faculty of Medicine, Hebrew University of Jerusalem, Jerusalem, Israel; 3 Imaging Department, Shaare Zedek Medical Center, Jerusalem, Israel; 4 Department of Pediatric Urology, Shaare Zedek Medical Center, Jerusalem, Israel; 5 Department of Urology, Shaare Zedek Medical Center, Jerusalem, Israel

**Keywords:** Emphysematous pyelonephritis, Infection-related calculi, Proteinaceous calculi.

## Abstract

Emphysematous pyelonephritis (EPN) is a critical and life-threatening necrotizing urinary tract infection, marked by gas formation within the renal parenchyma, collecting system or peri-nephric tissue and is extremely rare in children. Proteinaceous calculi are a rare type of kidney stones, reported in only 0.5% of nephrolithiasis cases, and rarely reported in the pediatric population. We present a case of a 16-year-old female with concurrent EPN and proteinaceous calculi with recurrent *Escherichia coli* urinary tract infection.

## Case presentation

A 16-year-old Caucasian female presented with vomiting and abdominal pain two days after initiating cefuroxime treatment for *Escherichia coli* pyelonephritis. Her medical history was unremarkable, aside from a previous episode of *Escherichia coli* (E. coli) pyelonephritis two months earlier, which was her first ever episode.

On examination she was afebrile (36.6 °C), tachycardic (150 bpm) and her blood pressure was 97/65 mmHg (< 5th percentile for age). Her height was 151.7 cm, weight 55 kg, BMI 23.9 (81.3%, normal BMI percentile is 5th–85th). Physical examination revealed pallor, abdominal and costovertebral angle tenderness.

**Fig. 1:**
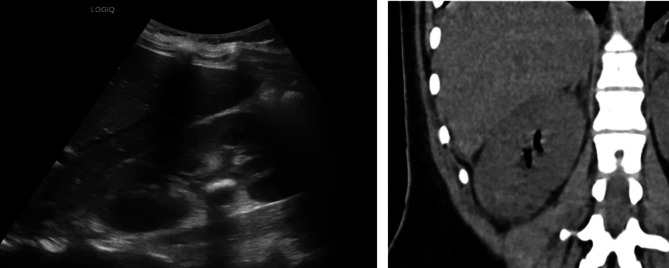
*Left:* Abdominal ultrasound demonstrating right kidney calculus, with minimal hydronephrosis. *Right*: Non-contrast computed tomography showing air in the collecting system of the right kidney, consistent with class 1 emphysematous pyelonephritis, defined as gas confined to the collecting system only [1]

Laboratory investigation demonstrated leukopenia (2.3, normal range (3.6–10) × 10^9^/L), normal platelets count (172 × 10^6^/L), plasma electrolytes were within normal range except for mild hyponatremia (134, normal range 135–145 mEq/L), minimal hyperglycemia (107, normal range 65–105 mg/dL), significantly elevated inflammatory markers (C-reactive protein 45.5 mg/dl (normal range 0–0.5 mg/dl), along with prolonged prothrombin time (18.9, normal range 10–14 s) and markedly increased D-dimer levels (4067, normal range 69–580 ng/ml). Additionally acute kidney injury was noted, with elevated plasma creatinine and decreased eGFR (1.11 mg/dl and 56.4 ml/min/1.73 m^2^ calculated using the Schwartz equation (normal range 0.5–0.9 mg/dl, 112 ± 13 ml/min/1.73 m^2^). Urinalysis was positive for leukocytes and nitrites. Taken together, the clinical and laboratory abnormalities were consistent with urosepsis. Therapeutic management included the initiation of ampicillin and gentamicin, in conjunction with repeated administration of intravenous fluids. Abdominal ultrasound demonstrated a calculus with minimal hydronephrosis. This finding was confirmed on non-contrast computed tomography (NCCT). Notably, an unexpected presence of air within the collecting system of the right kidney was observed, consistent with class 1 emphysematous pyelonephritis (EPN), defined as gas confined to the collecting system only [1] (Fig. 1).

Due to lack of clinical improvement and septic shock a 6/24 Fr double J stent was placed, resulting in the passage of turbid urine. The following day, she improved clinically, and her creatinine level normalized. Blood and urine cultures were positive for ampicillin-sensitive *E. coli*.

A week later, the patient underwent right ultrasound-guided-mini percutaneous-nephrolithotomy (PCNL). Kidney stone chemical analysis revealed mixed composition of calcium-oxalate-monohydrate (50%), protein (40%) and calcium-phosphate-carbonate (10%). A comprehensive metabolic evaluation for nephrolithiasis risk factors were negative, with no hyperoxaluria, hyperuricosuria or hypercalciuria. Citrate levels were within the normal range as well.

During a three-year follow-up period, the patient remained free of recurrent nephrolithiasis or pyelonephritis episodes.

## Discussion

Emphysematous pyelonephritis (EPN) is a life-threatening, necrotizing infection of the urinary tract characterized by gas accumulation within the renal parenchyma, collecting system or peri-nephric tissue. While well-documented in adults, EPN is exceedingly rare in the pediatric population, with a limited number of cases reported in the literature to date [2, 3]. Among adults, 95% of EPN cases occur in individuals with diabetes mellitus, with additional risk factors including urinary tract obstruction and immunodeficiency [4]. In contrast, reported pediatric cases typically occur in non-diabetic patients and are often associated with nephrolithiasis, obstructive uropathies and urinary tract infections [2, 3]. It is important to note that NCCT is not routinely performed in pediatric patients with nephrolithiasis, particularly in infants. Given that ultrasound is considerably less sensitive than CT in detecting intrarenal or perinephric gas, the true prevalence of EPN in children may be underestimated.

The pathophysiology of EPN is characterized by gas production resulting from mixed acid fermentation of glucose by gas-forming organisms, primarily members of the Enterobacteriaceae family, such as *Escherichia coli* and *Klebsiella pneumoniae*. Contributing factors include urinary tract obstruction, which elevates pelvicalyceal pressure and impairs renal perfusion, thereby favoring an anaerobic environment conducive to bacterial growth. These alterations also compromise the local delivery of immune cells and antibiotics, further promoting infection progression and gas accumulation within the renal parenchyma and adjacent structures [1]. Clinically, EPN presents with symptoms similar to severe pyelonephritis such as fever, tachycardia, costovertebral tenderness, flank pain and hematuria. Pneumaturia is a distinctive feature of EPN. Palpable crepitus is found in less than 10% of cases [4]. Acute kidney injury and bacteremia are common complications, whereas pneumomediastinum is rare. The reported mortality rate in EPN is 12.5% and often associated with shock, thrombocytopenia, confusion, hyponatremia and emergency nephrectomy [4]. Timely diagnosis and the early initiation of aggressive antibiotic therapy, in conjunction with urologic interventions such as ureteral stenting or percutaneous nephrostomy—as illustrated in our patient—are crucial for improving patient survival. Early diagnosis may also allow for a more conservative therapeutic approach, potentially obviating the need for partial or total nephrectomy in a subset of patients.

Our patient was also found to have a proteinaceous kidney stone, also known as matrix stone, which is an uncommon type of kidney stone, reported in only 0.5% of nephrolithiasis cases [5]. To our knowledge, only one pediatric case has been reported. A potential explanation for the limited number of reports may lie in the widespread use of attenuated total reflectance Fourier-transform infrared (ATR-FTIR) spectroscopy for the analysis of kidney stones. This technique generally identifies proteins as part of a broader"organic matrix"rather than quantifying them individually, which may contribute to the under-recognition of protein-rich stones in both clinical practice and the scientific literature [6]. Proteinaceous kidney stones are composed mainly of proteins with minimal crystalline mineral content, are noted for their soft and pliable nature which can be completely radiolucent in case of absence of crystalline constituents. Proteomic analyses have identified key inflammatory proteins, such as S100-A8 and S100-A9, as major components of matrix stones. The marked presence of these inflammatory molecules suggests that the inflammatory process is likely an initiating factor in the formation of soft calculi rather than as a consequence of such formation [7].

We propose that the presence of both EPN and proteinaceous calculus in our patient resulted from recurrent *E. coli* urinary tract infection*,* which promoted a favorable environment for the formation of proteinaceous stone, subsequently leading to increased bacterial load and EPN.

## Summary

To the best of our knowledge, this is the first pediatric case of EPN associated with proteinaceous calculus to be published. Recurrent *E. coli* infections prompted the occurrence of these two rare conditions.

### What is new?

- This is the first published pediatric case of EPN with proteinaceous calculus.

## Data Availability

The data that support the findings of this study are available from the corresponding author upon reasonable request.

## Declarations

All authors contributed to the study conception and design. Material preparation, data collection and analysis were performed by Shimrit Tzvi-Behr,, Efrat Ben-Shalom, Ruth Cytter-Kuint, Yaacov Frishberg, Boris Chertin, and Ilan Z Kafka. The first draft of the manuscript was written by Shimrit Tzvi-Behr and all authors commented on previous versions of the manuscript. All authors read and approved the final manuscript. As Corresponding Author, I confirm that the manuscript has been read and approved for submission by all the named authors.
